# Genomic test ends a long diagnostic odyssey in a patient with resistance to thyroid hormones

**DOI:** 10.1186/s13044-019-0068-y

**Published:** 2019-07-15

**Authors:** Todor Arsov, Chengmei Xie, Nan Shen, Dan Andrews, Carola G. Vinuesa, Olivija Vaskova

**Affiliations:** 1 China-Australia Centre for Personalised Immunology, Shanghai Renji Hospital, Shanghai Jioatong University, Shanghai, China; 2 Centre for Personalised Immunology, John Curtin School of Medical Research, Australian National University, Canberra, Australia; 3 Institute of Pathophysiology and Nuclear Medicine, University Clinical Hospital, Skopje, Macedonia

**Keywords:** Resistance to thyroid hormones, Hashimoto thyroiditis, Genomic testing, Whole exome sequencing

## Abstract

**Background:**

Resistance to thyroid hormones is a very rare condition, which is often misdiagnosed and mistreated. The cases where there is a concomitant autoimmune thyroid disorder are ultra-rare and particularly challenging to treat. Diagnostic and research-based genomic testing can sometimes identify pathogenic variants unrelated to the primary reason for testing (incidental findings).

**Case presentation:**

We present a patient with thyroid resistance associated with hypothyroid Hashimoto thyroiditis. The long diagnostic odyssey spanning over 20-years included repeated misdiagnoses and mistreatments and was concluded by a research-based genomic testing, identifying a “de novo” *THRB* pathogenic variant. The varying sensitivity of various tissues to thyroid hormones accompanied by hypothyroid Hashimoto thyroiditis continues to pose a significant treatment challenge.

**Conclusions:**

Thyroid hormone resistance continues to be an un(der)- and misdiagnosed thyroid condition whose management is particularly challenging when associated with autoimmune thyroid disease. Whole exome sequencing has the potential to identify *THRB* pathogenic variants as incidental findings. Reporting such secondary findings from genomic testing may be particularly important in the context of the rarity of the condition and the potential clinical consequences of misdiagnosis and mistreatment.

## Introduction

Resistance to thyroid hormones – RTH (OMIM 190160, 188570, 145650) is a rare condition characterised by increased levels of thyroid hormones (particularly free fractions, fT4, fT3) and inappropriately unsuppressed levels of TSH, often in the normal or even increased range [1–3]. Although the true prevalence of this condition is not known, it is considered to be a very rare condition affecting about 1 in 40000 people, with about 3000 patients described worldwide so far [1–3].

The classical RTH is an autosomal dominant condition due to either germline inherited or ‘de novo’ dominant negative pathogenic variants in the *THRB* gene (coding for the beta subunit of the thyroid hormone receptor, THRβ), rendering the thyroid receptor less responsive to the thyroid hormones [1–3]. Mosaic forms of RTH due to pathogenic variants in THRβ have also been described [4].

The variable expression of THRβ in various tissues resulting in varying degrees of relative thyroid deficit and the increased T4/T3 signalling through the unaffected THRα make it difficult to understand the genotype - phenotype relationship and to make sense of the laboratory results and interpret them in the context of the clinical features in RTH [5, 6]. The clinical features in RTH are highly variable, even within the same family harbouring the same THRβ pathogenic variant [5]. Most patients with RTH present with goitre and/or tachycardia, hyperactive behaviour, and about half of the patients have neurocognitive symptoms including emotional disturbances, anxiety, learning difficulty, developmental delay and intellectual disability. Most patients reach normal stature, but children can have lower body weight and height [1, 3, 7].

The syndrome of RTH, like other rare conditions, poses complex diagnostic and treatment challenges and can lead to long and expensive diagnostic odysseys and suboptimal patient care [8–10]. The diagnostic power of genomic testing based on next generation sequencing, has revolutionised the field of rare diseases [11, 12]. Notwithstanding its diagnostic utility, genomic testing also introduced some challenges, such as reporting of incidental (and/or secondary) findings, particularly in the context of genomic testing of children [13, 14]. Despite the ability of genomics to provide diagnosis in 20–60% cases, access to genetic services remains a major challenge in the developing low- and middle-income countries due to lack of genetic resources, infrastructure and expertise [15, 16].

We present a complex case of resistance to thyroid hormones and Hashimoto thyroiditis (HT) with a long diagnostic odyssey ended by genomic testing in a research setting and discuss the challenges of diagnosis and clinical management of this patient with RTH-related unusual thyroid hormones homeostasis combined with a HT-related hypothyroid state.

## Case report

The patient is the elder of two sisters in the family, born prematurely at 7-month gestation to non-consanguineous parents and had normal development. She presented for medical attention at age 19 with Jackson-type seizures and was referred for her first thyroid evaluation (6/91, Table 1) due to a history of nervousness, heat intolerance, mild sleep disturbance and heart palpitations. Clinically, she was tachycardic (96 bpm), with sweaty hands, slightly palpable thyroid gland, with no tremor and normal blood pressure. A diagnosis of hyperthyroidism was made based on elevated thyroid hormone levels, and positive antimicrosomal antibodies, despite the unsuppressed TSH levels and the patient was started on antithyroid treatment (6/91, Table 1). In the course of the following 3 years the patient was on and off antithyroid treatment due to exaggerated response to tiamazol (9/91, Table 1) with varying clinical symptoms consistent with hyperthyroid state (4/93). There was a suspicion of high levels of thyroid hormone carriers, which was ruled out by high fT4 levels (1/94, Table 1).

**Table 1:** Patient’s diagnostic and management odyssey

Date	Symptoms	T4 fT4	T3 fT3	TSH	TAB	Size	Treatment
6/91	nervous, palpitation, sweating	> 200 n/a	4.2 n/a	1.4	aMS+	N	start tiamazol
9/91	feeling better	< 47 n/a	n/a	n/a	n/a	2.5 N	reduced tiamazol
3/92	no symptoms	> 320 n/a	4.0 n/a	1.9	n/a	2.5 N	2 months no treatm ? high ThyH carriers
4/93	tremor, tachycardia, weight loss	> 340 n/a	n/a	2.1	n/a	n/a	start tiamazol
6/93	no change	279 n/a	n/a	n/a	n/a	n/a	discontinued ? high ThyH carriers
1/94	nervous tachycardia	257 38	n/a	1.4	n/a	n/a	start tiamazol
5/94	asymptomatic	173 n/a	n/a	n/a	n/a	n/a	discontinued
9/94	asymptomatic	228 46	2.2 n/a	1.2	n/a	n/a	metimazol
11/94	asymptomatic	206 28	2.2 n/a	1.4	n/a	n/a	discontinued ? ThyH resistance
9/96	asymptomatic	250 93	2.1 14	1.9	n/a	n/a	no treatment
	TRH test following 7 days T3 overload – normal TSH response (basal TSH 0.23, 30′ 2.1, 60′ 1.2, 90′ 0.89) DNA sent to overseas lab for genetic testing, returned no result.						
until 8/2007		to 184 to 79	to 3.2 to 9.5	to 2.7	aHTG 37.5 aTPO 1642	N firm	no treatment ? HT
until 12/2015	lost to follow up RA on MTX, bioTx	sought international thyroid expertise, diagnosed and treated for thyrotoxicosis due to elevated fT4, fT3 and despite unsuppressed TSH					
12/15	tired, slow weight gain	n/a 31.4	n/a 14.3	5.6	n/a	firm hyperECH	ThyH replacement Hypothyroid HT
2018	Patient recruited in an international study of genetics of autoimmunity and underwent whole exome sequencing providing genetic confirmation of the RTH (pathogenic variant in THB, p.Arg338Trp).						
2019							

Abbreviations and normal laboratory values: TRH – thyrotropin releasing hormone, RA – rheumatoid arthritis, MTH – methotrexate, T4 – total T4 (units μg/ml, normal 5–14.1), fT4 – free T4 (units pmol/L, normal 11–21), T3 – total T3 (units ng/ml, normal 0.8–2.1), fT3 – free T3 (units pmol/L, normal 3.1–6.0), TSH (units mU/ml, normal 0.4–4.3), ThyH – thyroid hormone(s), aMS – antimicrtosomal antibodies, aHTG – antihuman thyroglobulin antibodies, aTPO – antitissue peroxidase antibodies, hyperECH – hyperechogenic, HT – Hashimoto thyroiditis, RTH – resistance to thyroid hormones, THB – thyroid hormone receptor, beta subunit, n/a – not assessed

The first clinical suspicion of RTH was documented in 11/94 and corroborated by the high levels of total and free thyroid hormones and unsuppressed TSH, and a result from TRH test under suppression with high dose T3 consistent with RTH. The patient remained without treatment for the following 10-years, and was lost to follow up during the subsequent 8 years. During this period, the patient sought international thyroid expertise, and was diagnosed and treated for thyrotoxicosis based on high total and free thyroid hormone levels and some symptoms consistent with a hyperthyroid state, despite unsuppressed TSH levels. Of note, during this period she was diagnosed with a moderate to severe rheumatoid arthritis (RA) and treated with methotrexate and subsequently with biologic therapy.

In 12/15 (Table 1) the patient presented to our department complaining of feeling tired, slow and intolerant to cold, with levels of thyroid hormones lower and TSH level higher than her usual, elevated levels of cholesterol and weight gain of 17 kg over the preceding 8-year period. Those findings together with positive antithyroid antibodies, palpatory firm gland with non-homogenous hyperechogenic ultrasound appearance lead to the diagnosis of hypothyroid Hashimoto thyroiditis and she was initiated on thyroid replacement treatment.

**Fig. 1:**
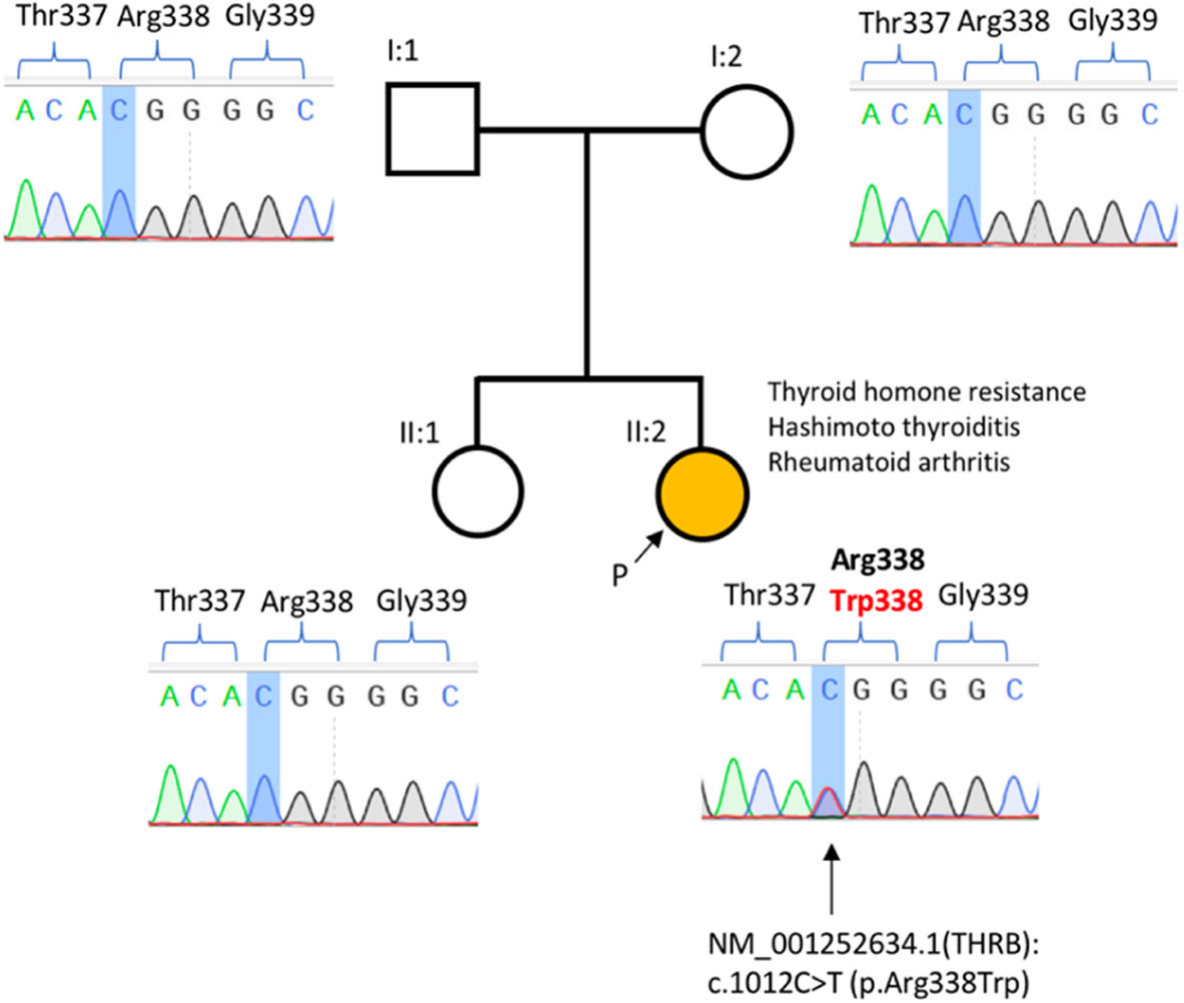
Patient’s pedigree and results from genetic testing. Whole exome sequencing identified a heterozygous “de novo” pathogenic variant NM_001252634.1(THRB):c.1012C > T changing evolutionary conserved amino acid Arg338 to Trp (p.Arg338Trp). The variant was not found in the healthy parents (I:1, I:2) and sister (II:1), both maternity and paternity was confirmed with microsatellite testing (data not shown)

In 2018 our patient was recruited in an international research study approved by the Human Genetic Committees of the Australian National University and The Canberra Hospital and underwent whole exome sequencing. The details of the used genomic and bioinformatic methodology is described elsewhere [17]. This testing identified a “de novo” pathogenic variant (with confirmed paternity and maternity, data not shown) in the beta subunit of the thyroid hormone receptor, *THRB* p.Arg228Trp) leading to RTH (Fig. 1). This variant has been described in more than 30 families/cases with RTH so far [18], and has not been described in the databases of human variation (dbSNP, 1000 genomes). The variant affects an evolutionary highly conserved amino acid, is predicted to be damaging/deleterious by in silico prediction tools (PolyPhen, SIFT), and in vitro studies have shown that this variant affects T3 binding to the thyroid hormone receptor [19].

The patient continues to be treated with low doses thyroid replacement therapy, mostly based on clinical features, taking into consideration her personal usual levels of thyroid hormones and TSH. The ongoing challenge is judging her thyroid state clinically given the variable distribution of alpha and beta thyroid hormone receptors leading to discrepant symptoms of hyperthyroidism (tachycardia, sweating) and hypothyroidism (hypercholesterolemia, weight gain, cold intolerance).

## Discussion

We present an unusual and rare case of rare RTH and hypothyroid HT. As other in other cases, the RTH in our case was initially misdiagnosed and mistreated as autoimmune thyrotoxicosis aiming to reduce thyroid hormone levels, which should be avoided particularly in pregnant patients [3, 20–23]. We would argue that the same holds true in (young) children with RTH, where antithyroid treatment could be dangerous and potentially detrimental to the intellectual development. Unfortunately, given no prior clinical experience with this very rare condition and lack of access to genetic testing services and clinical genetics support, the diagnosis of RTH in this patient was not confirmed until 24 years later. This long diagnostic odyssey contributed to patient’s mistrust and frequent change of thyroid specialists, which at some stage lead to the correct clinical diagnosis of thyroid hormone resistance, but also prompted the patient to seek second opinions from thyroid physicians, including international experts, which ultimately lead to repeated misdiagnosis and mistreatment with antithyroid medications.

Our case was made more challenging by the co-existence of HT, formally diagnosed later in the course of the disease. For a while, the thyroid autoimmune process in our patient was detectable only on level of positive thyroid autoantibodies and later it manifested with increasing level of thyroid autoantibodies, ultrasonographic changes consistent with inflammation and overt clinical hypothyroidism with increased levels of TSH (compared to the usual in this patient) - established the diagnosis of HT and prompted treatment with thyroid hormone replacement which is ongoing at this point in time [24]. The association between autoimmunity and RTH and the possible underlying mechanisms remain anecdotal [25] and there are isolated reports of both RTH associated with Graves disease [26–30] and Hashimoto thyroiditis [31–36] – all emphasising the diagnostic and management challenges posed by these associations, cautioning about the possibility of erroneous diagnosis and treatment.

Our case is unusual in that the genetic diagnosis was established by genomic testing, using whole exome sequencing. While the genetic test did not find a causative variant for the immune-mediated conditions in the patient (RA and HT), it detected a pathogenic *THRB* variant causing RTH. This finding could be considered an “incidental” finding in the context of the primary autoimmunity focus of our research project, and pose the dilemma of reporting an incidental finding for a condition (RTH) which affects the same organ (thyroid axis) as the primary condition (HT) [13]. This consideration prompted us to ask the question whether the *THRB* gene should be nominated for the list of reportable “actionable” incidental findings [13, 37]. In the context of increased use of genomic testing in children (including young children and even newborn babies), it is plausible that there may be instances where children with RTH and pathogenic *THRB* variants are sequenced for unrelated reasons, but would benefit greatly from having the information about having RTH, which could prevent misdiagnosis and potentially harmful consequences of iatrogenic hypothyroidism induced by treatment with antithyroid medications. We also wonder about the full applicability of the protocol used to assess the “clinical actionability” associated with genomic variation [37] in the context of such a rare disease as RTH, for which it may be a while before we have clinical practice guidelines, or high-level evidence from systemic reviews and/or meta-analyses.

## Conclusion

In conclusion, RTH is a very rare and often misdiagnosed and mistreated condition, often creating a long diagnostic odyssey due to lack of access to genetic services. When recognised RTH poses a significant treatment challenge, particularly when combined with a more common autoimmune thyroid disease. Elevated levels of thyroid hormones, particularly fT4 and fT3, with unsuppressed TSH levels should raise suspicion of RTH and prompt referral to a tertiary thyroid centre with capacity to differentiate and established this rare diagnosis.

## Data Availability

Please contact corresponding author for data requests.

## Abbreviations

(f)T3: (free) triiodothyronine
(f)T4: (free) thyroxine
HT: Hashimoto thyroiditis
RA: Rheumatoid arthritis
RTH: Resistance to thyroid hormones
THRB: Thyroid hormone receptor beta subunit gene
TSH: Thyroid stimulating hormone
